# Ivermectin: Panacea or true promise for COVID-19

**DOI:** 10.17179/excli2020-3022

**Published:** 2020-11-09

**Authors:** Luana Heimfarth, Victor Santana Santos, Adriano Antunes de Souza Araújo, Lucindo José Quintans-Júnior, Paulo Ricardo Martins-Filho

**Affiliations:** 1 Laboratory of Neuroscience and Pharmacological Assays (LANEF), Federal University of Sergipe, São Cristóvão, Brazil; 2 Centre for Epidemiology and Public Health, Federal University of Alagoas, Arapiraca, Brazil; 3 Department of Pharmacy, Federal University of Sergipe, São Cristóvão, Brazil; 4 Investigative Pathology Laboratory, Federal University of Sergipe, Aracaju, Brazil

## ⁯

***Dear Editor, ***

To date, there are no effective and specific treatments available against Severe Acute Respiratory Syndrome Coronavirus 2 (SARS-CoV-2) and the medical protocols include isolation measures of the patients and treatment of symptoms. Instead of developing new compounds against SARS-CoV-2 that could take years to be approved, researchers have sought to redirect safety drugs already approved for other diseases. Controversially, a chasm has opened up between scientific information about drugs to coronavirus disease (COVID-19) outbreak and the speed that physicians need more information thereupon, thus entering in the use of medications that are more doubtful than certain. Moreover, if new compounds are discovered, geopolitical and economic characteristics will determine their use to communities. Therefore, it would be ideal to find a low-cost and accessible drug for COVID‐19. In this context, the ivermectin emerges as a potential treatment option for SARS-CoV-2 infection (Peña-Silva et al., 2020[12]) to help physicians and patients to cross the valley of death imposed by COVID-19 or succumbing to it?

Ivermectin is a safe and effective FDA-approved macrocyclic lactone with a broad-spectrum antiparasitic pharmacological activity (González Canga et al., 2008[5]). It causes stimulation of gamma amino butyric acid (GABA)-gated-Cl^−^ channels, leading to hyperpolarization and consequently blocking neurotransmission in neurons and myocytes, resulting in paralysis and death of the infesting organism (Geary, 2005[4]). Regarding ivermectin role as an antiviral agent, a recent systematic review shows that this antiparasitic drug seems to be highly effective against some viruses *in vitro* (Heidary and Gharebaghi, 2020[6]) including West Nile virus (Yang et al., 2020[17]), HIV-1, dengue virus (Wagstaff et al., 2012[15]), yellow fever virus (Mastrangelo et al., 2012[10]), Chikungunya virus (Varghese et al., 2016[14]) and Venezuelan equine encephalitis virus (Lundberg et al., 2013[8]). However, studies on the antiviral efficacy of ivermectin in animal models are few and ambivalent, showing to be favorable and unfavorable against pseudorabies (Lv et al., 2018[9]) and Zika virus (ZIKV) (Ketkar et al., 2019[7]), respectively. 

The antiviral profile of ivermectin is related to the inhibitory effect of this drug on importin (IMPα/β1) heterodimer-mediated nuclear import of viral proteins, which is a crucial transporter in nucleocytoplasmic shuttling (NS) of the SARS-CoV nucleocapsid protein and indispensable for viral replication (Wagstaff et al., 2012[15]; Caly et al., 2020[2]). Caly et al. (2020[2]) demonstrate that ivermectin (5 μM) has antiviral action against the SARS-CoV-2 *in vitro*, with a single dose able to control viral replication within 24-48 hours. Some authors proposed that the inhibition of NS in the early course of infection induced by ivermectin could attenuate the severity, duration and spread of the infection (Caly et al., 2020[2]).

Moreover, existing literature suggested potential role of ivermectin in treating viral infection complications due to its capacity in suppressing the cytokine production (Yan et al., 2011[16]) and decrease the transcription of oxidative and inflammatory mediators by inhibiting NFκB activation in rodents (Zhang et al., 2008[18]). Therefore, the successful *in vitro* inhibitory effect of ivermectin against several RNA virus and the influence in host immune responses warrant further studies to establish its potential role in human COVID-19 infections. 

After the confirmation of antiviral activity of ivermectin against SARS-CoV-2 virus *in vitro*, several clinical trial protocols have been registered in the ClinicalTrials.gov evaluating the effects of this drug in combination with others treatment protocols in the COVID-19 therapy. A prospective, multi-center, randomized, double-blind trial study enrolling 102 participants was designed to assess the efficacy and safety of ivermectin (600 μg/Kg or 1200 μg/Kg) for the treatment of initial infection with SARS-CoV2 in mild and moderate patients (NCT04438850; Bisoffi, 2020[1])). In addition, a clinical trial has tried to prove the potential of ivermectin as a prophylaxis drug in viral infection. In that interventional study, asymptomatic family close contact of confirmed COVID-19 patient receives prophylactic ivermectin and the symptoms for diagnosis of COVID-19 are monitored for 14 days (NCT04422561; Shouman, 2020[13]). Finally, some studies used the drug combination as pharmacological option. In this context, a randomized, doubled-blind and placebo-controlled phase II research enrolled 176 patients with diagnosis of active cancer to evaluate the efficacy of the early use of ivermectin plus losartan in cancer patients' recent diagnosis of COVID-19 (NCT04447235; Exman, 2020[3]). However, to date, the preliminary results have not yet been reported.

In the last months, due to the favorable results of ivermectin as antiviral drug *in vitro*, the spread use of this drug become a panacea in South America, especially stimulated by a scientific denialism or even by the current chasm to a gold standard treatment for COVID-19. On May, ivermectin was included as a COVID-19 treatment in Peru and Bolivia and on June 2020, at least one municipality in Brazil had approved the use of ivermectin as a preventive drug for COVID-19 (Offord, 2020[11]). 

Although *in vitro* evidence showed that ivermectin is able to control SARS-CoV-2 virus replication (Caly et al., 2020[2]), the *in vivo* studies reporting its antiviral activity present contradictory findings (Lv et al., 2018[9]; Ketkar et al., 2019[7]) . Moreover, the controlled clinical trials evaluating the safety and efficacy of ivermectin as a potential antiviral treatment of COVID-19 are still lacking. Thus, high-quality trial evidence is necessary to use ivermectin in the management of SARS-CoV-2 infection, as well as to prove its efficacy as prophylactic drug; the scientific evidence being to be the bedrock for its use and not the unbridled desire for an improbable cure coming from a “miracle drug”.

## Acknowledgment

Authors thank FAPITEC-SE, CAPES and CNPq. This study is part of EpiSERGIPE project.

## Disclosures

No conflicts of interest, financial or otherwise, are declared by the authors.

## Authors’ contributions

P.R.M.F, V.S.S, L.H, A.A.S.A and L.J.Q.J drafted manuscript; P.R.M.F, V.S.S, L.H, A.A.S.A and L.J.Q.J edited and revised the manuscript; P.R.M.F, V.S.S, L.H, A.A.S.A and L.J.Q.J approved the final version of manuscript.
